# Dipsacoside B Attenuates Atherosclerosis by Promoting Autophagy to Inhibit Macrophage Lipid Accumulation

**DOI:** 10.3390/biom14101226

**Published:** 2024-09-27

**Authors:** Wenjuan Quan, Taoli Sun, Bo Hu, Quanye Luo, Yancheng Zhong, Wen Chen, Qinhui Tuo

**Affiliations:** 1 Key Laboratory for Quality Evaluation of Bulk Herbs of Hunan Province, School of Pharmacy, Hunan University of Chinese Medicine, Changsha 410208, China; quanwenjuan@stu.hnucm.edu.cn (W.Q.); suntaoli@stu.hnucm.edu.cn (T.S.); 2 Department of Critical Care Medicine, Changde Hospital of Hunan University of Chinese Medicine, Changde 415000, China; 3 Key Laboratory of Vascular Biology and Translational Medicine, Medical School, Hunan University of Chinese Medicine, Changsha 410208, China; hubo@zzu.edu.cn (B.H.); 20223777@stu.hnucm.edu.cn (Q.L.); zhongyancheng@hnucm.edu.cn (Y.Z.)

**Keywords:** dipsacoside B, atherosclerosis, autophagy, foam cells, *Lonicerae flos*, Shanyinhua

## Abstract

Atherosclerosis is a chronic inflammatory disease characterized by lipid accumulation and foam cell formation in the arterial wall. Promoting macrophage autophagy has emerged as a promising therapeutic strategy against atherosclerosis. Dipsacoside B (DB) is an oleanane-type pentacyclic triterpenoid saponin extracted from *Lonicerae flos* with potential anti-atherosclerotic properties. In this study, we investigated the effects of DB on atherosclerosis progression in ApoE^−/−^ mice fed a high-fat diet and explored the underlying mechanisms in oxidized low-density lipoprotein (ox-LDL)-induced foam cells. DB treatment significantly reduced atherosclerotic lesion size, improved plaque stability, and regulated lipid metabolism without impairing liver and kidney function in ApoE^−/−^ mice. In vitro studies revealed that DB dose-dependently inhibited ox-LDL internalization and intracellular lipid accumulation in RAW264.7 macrophages. Mechanistically, DB induced autophagy, as evidenced by increased autophagosome formation and upregulated expression of autophagy markers LC3-II and p62 both in vivo and in vitro. Inhibition of autophagy by chloroquine abolished the antiatherosclerotic and pro-autophagic effects of DB. Furthermore, DB treatment increased LC3-II and p62 mRNA levels, suggesting transcriptional regulation of autophagy. Collectively, our findings demonstrate that DB exerts anti-atherosclerotic effects by inhibiting foam cell formation via autophagy induction, providing new insights into the pharmacological actions of DB and its potential as a therapeutic agent against atherosclerosis.

## 1. Introduction

Atherosclerosis (AS), typically arising in large and medium arteries, is a degenerative process in which fatty plaque accumulates in the arterial walls, eventually leading to vascular stenosis or the development of susceptible plaques as the illness progresses. Initially, patients exhibit no obvious symptoms, but as vascular stenosis worsens and susceptible plaques rupture, heart attacks, strokes, deep vein embolisms, and hemangiomas can occur [1]. According to Global Burden of Disease Study statistics [2], cardiovascular disease (CVD) is the leading cause of death worldwide, and AS is associated with approximately 80% of cardiovascular disease, contributing to 18 million deaths annually. Approximately 180 million people have AS-related cardiovascular disease, among which China has recently been estimated to have 270 million AS sufferers [3]. The burden of atherosclerotic cardiovascular disease (ASCVD) is increasing at a rapid and significant rate, with both the incidence and mortality of ASCVD rising annually. These trends are critical aspects of the epidemiology of cardiovascular diseases in China. ASCVD has become the leading cause of death, endangering the health of the country and placing a heavy strain on its healthcare system [4,5,6].

Clinically, lipid-lowering, antiplatelet, anticoagulant, thrombolytic, vasodilatory, antioxidant, and anti-inflammatory medications are potent strategies for the treatment of AS [7]. Despite the efficacy of these treatments, there is a risk of side effects, including abnormal liver function [8], gastrointestinal bleeding [9], and restenosis following percutaneous coronary intervention (PCI) [10]. Consequently, there is an urgent need for proactive and effective combined therapies that can minimize unpleasant side effects and produce a synergistic effect.

According to traditional Chinese medicine theories, there are three main pathogenic elements, ‘phlegm’, ‘blood stasis’, and ‘poison’, that contribute to AS [11,12,13]. ‘Blood stasis’ causes ‘phlegm’, which eventually turns into ‘poison’. Thus, the primary pathophysiology of AS is the accumulation of heat and toxin. Recent studies have demonstrated that according to this pathogenesis, the application of heat-clearing and toxic-detoxification prescriptions, such as Huang-Lian-Jie-Du decoction [14], Si-Miao-Yong-An decoction [15], and San-Huang-Xie-Xin decoction [16], can produce significant curative effects on AS-related diseases. Recent research has shown that monomers derived from traditional Chinese herbs with cleansing and detoxifying properties, like berberine [17], emodin [18,19] and gypenoside [19], are effective in preventing the onset and progression of AS. All the evidence indicates that the heat-clearing and detoxicating method is an excellent remedy for AS.

*Lonicerae flos* (Shanyinhua in Chinese), encompassing four species [20]—*Lonicera macranthoides* Hand.-Mazz., *Lonicera confusa* (Sweet) DC., *Lonicera hypoglauca* Miq., and *Lonicera fulvotomentosa* P.S. Hsu et S.C. Cheng—is a Chinese herbal medicine that is used to dispel heat and promote detoxification [20,21]. Studies have demonstrated that these species possess significant anti-inflammatory [22] and antioxidant properties [23]. Additionally, *Lonicera macranthoides* Hand.-Mazz. [24] has been shown to significantly reduce the area of AS plaques and the plaque-to-wall area ratio, alleviate AS changes in ApoE^−/−^ mice, and decrease lipid droplets and cholesterol concentrations in lipid-loaded THP-1 macrophages. These combined actions make *Lonicerae flos* a valuable herbal remedy in the prevention and management of AS.

Dipsacoside B (DB) is a kind of oleanane-type pentacyclic triterpenoid saponin extracted from *Lonicerae flos*. Recent studies have shown that DB protects against liver damage caused by acetaminophen [25] and reduces brain damage in rats with ischemic stroke by inhibiting the activity of mitochondrial E3 ubiquitin ligase 1 [26]. According to our earlier research [27], DB exhibits good vascular activity by preventing Vascular Smooth Muscle Cell (VSMC) migration and proliferation, as well as the development of neointima in rats with balloon injury. The actions of oleanane-type saponins, such as anti-obesity [28], lipid-lowering [29], anti-AS [30], blood pressure-lowering [31], and blood glucose-reducing [32], indicate that these compounds have metabolic regulatory effects. There is a conjecture that DB possesses strong pharmacological efficacy against AS due to its comparable chemical structure.

Autophagy is a protective mechanism for cells to achieve energy recovery and self-renewal through lysosomal degradation of waste proteins or damaged organelles [33]. Studies have demonstrated that promoting macrophage autophagy [34] significantly slows the progression of AS. Pentacyclic triterpene oleanane-type saponins, such as Aralia saponin C [35], natural saponin EsC [36], and the total glycoside of celosia seed [37], can act on autophagy and have therapeutic effects. DB plays a protective role in the liver through the autophagy pathway [25]. However, whether DB exerts anti-AS effects through autophagy has not been reported.

In the present study, we used ApoE^−/−^ mice to evaluate the attenuation effects of DB on AS lesions in vivo. We also examined DB’s role in the reduction in foam cells and investigated its potential interactions with autophagy. Our research will reveal the preventive effects of DB on AS-related diseases.

## 2. Materials and Methods

### 2.1. Animals and Treatment Protocols

Thirty-two male ApoE knock-out mice (ApoE^−/−^ mice) and eight male C57BL/6J mice (all aged 6 weeks), purchased from Beijing HFK Bio-Science Co., Ltd. (Beijing, China) were housed in a dedicated pathogen-free facility under controlled temperature (25 ± 1 °C) and humidity (55 ± 15%), with 12 h light-dark cycles and free access to food and water. After one week of adaptive feeding under standard animal housing conditions, ApoE^−/−^ mice (aged 7 weeks) were fed a high-fat diet (HFD, Beijing HFK Bio-Science Co., Ltd., Beijing, China) containing 77.5% basic mice maintaining food, 20% fat, 2% cholesterol, and 0.5% sodium cholic acid for 12 weeks to stimulate AS plaques. After being on HFD for 4 weeks, ApoE^−/−^ mice (aged 11 weeks) were subsequently divided into 4 groups and received corresponding therapy based on HFD for the following 8 weeks: HFD group receiving daily oral administration of normal saline, HFD + AT group receiving daily oral administration of atorvastatin (AT, A121956-1g, Shanghai Aladdin Biochemical Technology Co., Ltd., Shanghai, China) at a dose of 10 mg/kg, HFD + DB_L_ group receiving daily oral administration of DB (>98% purity by HPLC, M127897-10g, Beijing MREDA Technology Co., Ltd., Beijing, China) at a dose of 10 mg/kg, and HFD + DB_H_ group receiving daily oral administration of DB at a dose of 100 mg/kg. All of the C57BL/6J mice in Ctrl group were received homologous saline by gastric gavage for 8 weeks together with chow diet (CD, Beijing HFK Bio-Science Co., Ltd., Beijing, China). At the end of the experiment, the mice were sacrificed under anesthesia, and tissue samples were collected for further analysis.

### 2.2. Biochemical Examination

Blood samples were centrifuged at 3000 rpm for 15 min to collect serum. Serum levels of total cholesterol (TC), triglyceride (TG), LDL cholesterol (LDL-C), high-density lipoprotein (HDL), alanine aminotransferase (ALT), aspartate aminotransferase (AST), creatinine (CREA), and UREA were measured using an automatic biochemical analyzer (Chemray 240, Rayto Life, Guangzhou, China) according to manufacturer’s protocol.

### 2.3. AS Lesion Analysis

After the last administration at the end of the 12th week, all the mice were euthanized, and the aortas were perfused with phosphate-buffered saline (PBS, PB180327, Wuhan Procell Life Science &Technology Co., Ltd., Wuhan, China). After being fixed in 4% paraformaldehyde (PFA, BL539A, Biosharp, Beijing Labgic Technology Co., Ltd., Beijing, China) for 72 h, the aortas were cut longitudinally and stained with 1% Oil Red O (ORO) solution (O0625-25G, Sigma, St. Louis, MO, USA). Meanwhile, cryosections of the aortic root, aortic arch, and abdominal aorta were prepared and stained with 1% ORO solution. The quantification of plaque area was carried out using Image-Pro Plus 6.0 software.

### 2.4. Histological Examination

For histological examination, aortic root and visceral organ samples such as the heart, lung, liver, spleen, and kidney were carefully isolated, fixed in 4% PFA, and embedded in paraffin to prepare paraffin sections at a thickness of 5 μm. The sliced sections of visceral organs were stained with hematoxylin and eosin (HE) or ORO solution. Additionally, the sliced sections of the aortic root were stained by HE (S191003, Wuhan Pinofi Biotechnology Co., Ltd., Wuhan, China) and Masson’s trichrome (S191015, Wuhan Pinofi Biotechnology Co., Ltd., Wuhan, China). Furthermore, immunohistochemical (IHC) staining was performed to detect the expression levels of cluster of differentiation 68 (CD68, 1:400, BS3638, Boster, Wuhan, China) and matrix metallopeptidase 9 (MMP-9, 1:400, GB11132, Servicebio, Wuhan, China) in the sliced sections of aortic root. All the slices were scanned by an automatic scanning system (BA600-4, Motic, Xiamen, China) and quantified using Image-Pro Plus 6.0 software.

### 2.5. Cell Culture and Treatment

RAW264.7 cells, provided by Cell Bank of the Chinese Academy of Sciences, were cultured in Dulbecco’s Modified Eagle Medium (DMEM, PM150210, Procell, Wuhan, China) supplemented with 10% fetal bovine serum (FBS, 13011-8611, EVERY GREEN, Hangzhou, China) and 1% penicillin-streptomycin (PB180120, Procell, Wuhan, China) in an atmosphere of 5% CO_2_ and at a temperature of 37 °C. The cells were preincubated with DB at concentrations of 10, 30, and 100 μM for 2 h. Then, oxidized low-density lipoproteins (ox-LDL, YB-0002, Yiyuan Biotech, Guangzhou, China) were added into the cell culture medium to a final concentration of 100 μg/mL for 48 h. For the autophagy inhibition experiment, the cells were preincubated with chloroquine (CQ, T8689, Shanghai Topscience Co., Ltd., Shanghai, China) at a concentration of 20 μM and then co-incubated with DB and ox-LDL as previously described.

### 2.6. Cell Viability Assays

RAW264.7 cells were seeded in 96-well plates at a density of 5 × 10^3^ cells per well. After treatments with different concentrations of DB for 24 h, 48 h or 72 h, cell viability was assessed by cell counting kit-8 (cck-8, BS350B, Biosharp, Beijing Labgic Technology Co., Ltd., Beijing, China) according to the manufacturer’s protocol.

### 2.7. ORO Staining

RAW264.7 cells were fixed in 4% PFA and then rinsed with PBS. RAW264.7 cells were stained with a 1% ORO solution to visualize intracellular lipid droplets for 30 min at 37 °C. Next, 60% isopropanol (I112011-4L, Shanghai Aladdin Biochemical Technology Co., Ltd., Shanghai, China) was used to remove the excess ORO solution on cells. After washing with PBS, the cells were photographed under an optical microscope and quantified using Image-Pro Plus 6.0 software.

### 2.8. Analysis of Dil-ox-LDL Uptake

1,10–Dioctadecyl-3,3,30,30-tetramethylindocarbocyanine perchlorate-labeled ox-LDL (Dil-ox-LDL, YB-0010, Yiyuan Biotech, Guangzhou, China) was used to study the scavenger receptor-mediated endocytosis of ox-LDL by RAW264.7 cells. After different treatments, the cells were incubated with 40 μg/mL Dil-ox-LDL for 4 h at 37 °C in the dark. The nucleus was stained with 4’,6-diamidino-2-phenylindole (DAPI, F6057, Sigma, St. Louis, MO, USA). Confocal laser scanning microscope (CLSM, Nikon A1, Tokyo, Japan) was used to acquire fluorescence images at 565 nm, and Image-Pro Plus 6.0 software was used to quantify the average fluorescence intensity.

### 2.9. Western Blotting Analysis

After treatments, the cell lysates were obtained in prechilled RIPA buffer (CW2333S, Jiangsu Cowin Biotech Co., Ltd., Taizhou, China) containing a cocktail of protease and phosphatase inhibitors (CW2200S, CW2383S, Jiangsu Cowin Biotech Co., Ltd., Taizhou, China). Protein samples (30 μg) were separated by 10% or 15% SDS-PAGE, transferred onto PVDF membranes (10600021, Cytiva, Boston, MA, USA), and blocked in 5% skimmed milk (1172GR500, BioFroxx, Guangzhou Saiguo Biotech Co., Ltd., Guangzhou, China) for 2 h at 37 °C. Then, the membranes were incubated with specific primary antibodies (LC3A/B: 1:1000, 12741T, CST, Danvers, MA, USA; P62: 1:2000, ab56416, Abcam, MA, USA) at 4 °C overnight. After washing, the membranes were incubated with corresponding secondary antibodies (1:10,000, Jackson Labs, Bar Harbor, ME, USA) for 1 h at 37 °C. Finally, the immunoreactive bands were detected by the enhanced chemiluminescence method using ChemiDoc^TM^ XPS + Imaging system (Bio-rad, Hercules, CA, USA). The protein bands were quantified using Image J v1.44 software and normalized to GAPDH (60004-1-Ig, 1:10,000, Proteintech, Wuhan, China).

### 2.10. Quantitative Real-Time PCR (qRT-PCR)

Total RNAs were isolated using TRIzol reagent (15596026, Invitrogen, Carlsbad, CA, USA) according to the manufacturer’s instructions and quantified by Nanodrop 2000 (Thermo Fisher Scientific, Waltham, MA, USA). The obtained RNAs were then reverse-transcribed into cDNA using 2× Taq Master Mix (P111-01, Vazyme, Nanjing, China) on T100^TM^ Thermal Cycler (Bio-Rad, Hercules, CA, USA). qRT-PCR was carried out with cDNA in triplicate using ChamQ Universal SYBR qPCR Master Mix (Q711-02, Vazyme, Nangjing, China) on a real-time PCR system (LightCycler^®^ 96, Roche Life Science, Waltham, MA, USA). The amplification conditions for qRT-PCR were as follows: a. preincubation: 95 °C for 30s. b. 3 step amplification: 95 °C for 10 s, 60 °C for 30 s, 72 °C for 30 s, 45 cycles. c. cooling: 37 °C for 30 s. d. melting: 95 °C for 10 s, 65 °C for 60 s, 97 °C for 1 s. β-Actin was used as a reference to normalize the data. Data were analyzed using the 2^−∆∆ct^ method. All primers used are listed in Table 1.

### 2.11. Transmission Electron Microscopy (TEM)

The treated cells were fixed with 2.5% glutaraldehyde (P1126, Solarbio, Beijing, China). After being submitted to ethanol dehydration, the cells were embedded in SPI-Pon™ 812 epoxy resin monomer (90529-77-4, SPI, West Chester, PA, USA). Ultrathin slices were prepared by an Ultrathin Slicer (EM UC7, Leica, Vizra, Germany) and stained with 2% uranyl acetate followed by 2.6% lead citrate. All grids were viewed and photographed using a TEM (HT7800, Hitachi, Tokyo, Japan) at 200 kV.

### 2.12. Immunofluorescence (IF) Staining

Serial sections of the aortic root were used for immunofluorescence staining. The slides were put in an oven at 60 °C for 2 h, followed by dewaxed in an environment-friendly dewaxing solution (G1128, Servicebio, Wuhan, China) and hydrated in gradient concentrations of ethanol (100%–95%–85%–60%). After antigen retrieval procedures, the slides were permeabilized in 0.3% Triton X-100 (T8200, Solarbio, Beijing, China) at 37 °C for 1.5 h, blocked in 5% bovine serum albumin (BSA, 4240gr100, BioFroxx, Guangzhou Saiguo Biotech Co., Ltd., Guangzhou, China) at 37 °C for 1.5 h, and incubated with P62 (1:2000, ab56416, Abcam, MA, USA) and LC3A/B (1:1000, 12741T, CST, Danvers, MA, USA) primary antibodies at 4 °C overnight. After washing, the slides were incubated with relevant secondary antibodies (1:1000, ab150081, ab150120, Abcam, MA, USA) at 37 °C for 1.5 h. The nuclei were stained with antifade mounting medium with DAPI (F6057, Sigma, St. Louis, MO, USA). Images were obtained using CLSM and analyzed using Image-Pro Plus 6.0. software.

RAW264.7 cells were seeded onto rounded glass slides matched to 24-well plates. After the indicated treatments, cells were fixed with 4% PFA, permeabilized with 0.3% Triton X-100, blocked in 5% BSA, incubated with P62 and LC3A/B primary antibodies at 4 °C overnight, and then incubated with relevant secondary antibodies. Cells were observed under CLSM and photographed. Image J software was used to analyze the fluorescence intensity.

### 2.13. Statistical Analysis

All results obtained are presented as mean ± SD based on 3 independent experiments. GraphPad Prism software version 8.3.0 was used for statistical analysis. Differences among treatment groups were assessed with one-way ANOVA, followed by Dunnett’s test for post hoc comparison when applicable. Statistical significance was defined as *p* < 0.05.

## 3. Results

### 3.1. DB Protects against AS Lesions in HFD-Induced ApoE^−/−^ Mice

**Figure 1:**
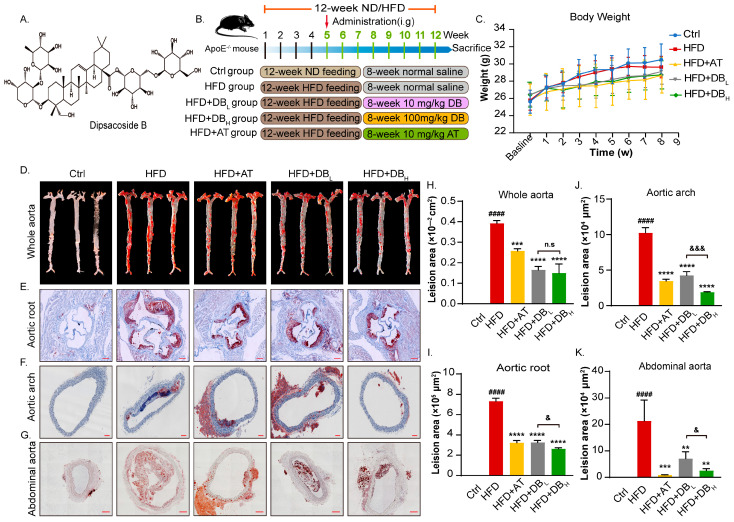
DB treatment efficiently inhibited the progression of AS in ApoE^−/−^ mice. (**A**) Chemical structure of DB. (**B**) Schematic illustration of animal experimental design. (**C**) Body weight of each administered group. (**D**,**H**) Representative images and quantitative analysis of lesion area in the aortas stained with ORO. (**E**,**I**) Representative cryosection images of aortic root stained with ORO and the corresponding quantitative analysis, 55× magnification, scale bar: 200 μm. (**F**,**J**). Representative cryosection images of aortic arch stained with ORO and the corresponding quantitative analysis, 100× magnification, scale bar: 100 μm. (**G**,**K**) Representative cryosection images of abdominal aorta stained with ORO and the corresponding quantitative analysis, 100× magnification, scale bar: 100 μm. Data are presented as mean ± SEM. vs. Ctrl group, ^####^ *p* < 0.0001. vs. HFD group, ** *p* < 0.01, *** *p* < 0.001, **** *p* < 0.0001. vs. HFD + DB_L_ group, n.s: no significance, ^&^ *p* < 0.05, ^&&&^ *p*< 0.001.

To determine the attenuation effects of DB (chemical structure shown in Figure 1A) on the development of AS lesions in HFD-induced ApoE^−/−^ mice, the mice were treated with DB for 8 weeks along with HFD to accelerate the process of atherogenesis (Figure 1B). During the experiment, no abnormal deaths were observed in any of the groups. Compared with the Ctrl group, ApoE^−/−^ mice fed with HFD did not show significant differences in body weight. Since week 3, the weight gain of mice treated with AT, DB_L_, and DB_H_ slowed down but showed no discernible difference among these groups. At the end of the 8-week treatment, there was no significant difference in body weight among the DB-treated groups (Figure 1C). We then examined the AS lesions formation by en face ORO staining, together with the same examination in areas, including the aortic root, aortic arch, and abdominal aorta. Results showed that aortic intima was intact and smooth in the Ctrl group, while notable AS lesions were observed in the HFD group. Smaller plaque areas were observed in the aforementioned areas after 8 weeks of DB and AT treatment in comparison with the HFD group, especially in the abdominal aorta sections. High-dose DB treatment showed a more potent effect on reducing atherosclerotic lesions (Figure 1D–K).

### 3.2. DB Promotes Plaque Stability in HFD-Induced ApoE^−/−^ Mice

**Figure 2:**
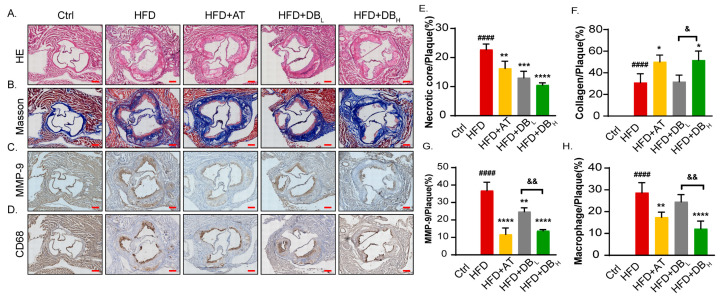
DB treatment prevented plaque development in ApoE^−/−^ mice. (**A**,**E**) Representative images and quantitative analysis of necrotic core in aortic roots stained with HE, 60× magnification, scale bar: 100 μm. (**B**,**F**) Representative images and quantitative analysis of necrotic core in aortic roots stained with Masson’s trichrome, 60× magnification, scale bar: 100 μm. (**C**,**G**) Immunohistochemistry staining with antibodies of MMP-9 and the corresponding quantitative analysis, 60× magnification, scale bar: 100 μm. (**D**,**H**) Immunohistochemistry staining with antibodies of CD-68 and the corresponding quantitative analysis, 60× magnification, scale bar: 100 μm. Data are presented as mean ± SEM. vs. Ctrl group, ^####^ *p* < 0.0001. vs. HFD group, * *p* < 0.05, ** *p* < 0.01, *** *p* < 0.001, **** *p* < 0.0001. vs. HFD + DB_L_ group, ^&^ *p* < 0.05, ^&&^ *p* < 0.01.

The assessment of plaque components is critical for the development of AS. As shown by HE staining images, notable necrotic regions were observed in the plaques of the HFD group. Treatment with AT, DB_L_, and DB_H_ led to a gradual decrease in necrotic core size, with DB_H_ showing the most pronounced effect (Figure 2A,E). Masson staining was employed to track the collagen content in the plaques. As anticipated, the DB treatment dramatically and dose-dependently raised the amount of collagen in the plaques (Figure 2B,F). Additionally, it was shown that in the plaques of ApoE^−/−^ mice, DB treatment inhibited the expression of MMP-9 (Figure 2C,G), the principal collagen-degrading enzyme of plaques, and CD68 (Figure 2D,H), the marker of macrophages.

### 3.3. DB Reduces Circulating Lipid Level and Hepatic Lipid Deposition

**Figure 3:**
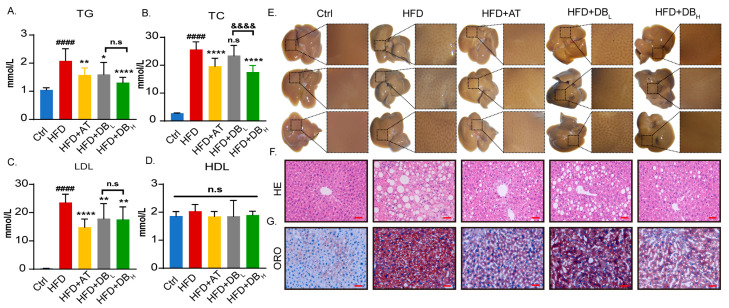
DB treatment regulated lipid metabolism in vivo. (**A**) The TG level in the serum of mice treated with different therapies. (**B**) The TC level in the serum of mice treated with different therapies. (**C**) The LDL level in the serum of mice treated with different therapies. (**D**) The HDL level in the serum of mice treated with different therapies. (**E**) Representative images of gross liver from different treated groups. (**F**) Representative images of hepatic tissue stained by HE, 200× magnification, scale bar: 50 μm. (**G**) Representative images of hepatic tissue stained by ORO, 200× magnification, scale bar: 50 μm. Data are presented as mean ± SEM. vs. Ctrl group, ^####^ *p* < 0.0001. vs. HFD group, * *p* < 0.05, ** *p* < 0.01, **** *p* < 0.0001. vs. HFD+DB_L_ group, n.s: no significance, ^&&&&^ *p* < 0.0001.

Lipid metabolism dysfunction is an independent risk factor for AS. HFD significantly increased the levels of TG, TC, and LDL-C in ApoE^−/−^ mice, but did not alter HDL levels (Figure 3A–D). As we expected, DB_H_ significantly reduced the levels of TG, TC, and LDL-C, while DB_L_ showed little effect on TC levels. We then examined lipid deposition in the liver. Results showed that DB treatment significantly reduced the yellow-white particles on the surface of the liver (Figure 3E). HE staining results indicated that DB reduced lipid droplets with empty vacuoles (Figure 3F). ORO staining showed lipid droplets in red, further confirming that DB treatment reduced the lipid content in hepatocytes (Figure 3G).

### 3.4. DB Exerts Its Role without Interfering with Liver and Kidney Function as Well as Organ Morphology

**Figure 4:**
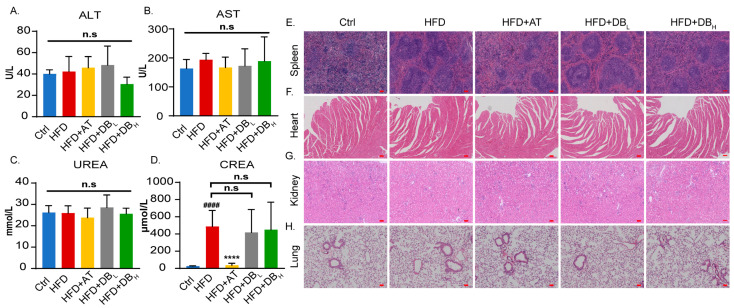
DB treatment demonstrated good drug safety. (**A**) The ALT level in the serum of mice treated with different therapies. (**B**) The AST level in the serum of mice treated with different therapies. (**C**) The UREA level in the serum of mice treated with different therapies. (**D**) The CREA level in the serum of mice treated with different therapies. (**E**) Representative images of splenic tissue stained by HE, 60× magnification, scale bar: 200 μm. (**F**) Representative images of cardiac tissue stained by HE, 60× magnification, scale bar: 200 μm. (**G**) Representative images of renal tissue stained by HE, 60× magnification, scale bar: 200 μm. (**H**) Representative images of lung tissue stained by HE, 60× magnification, scale bar: 200 μm. Data are presented as mean ± SEM. vs. Ctrl group, ^####^ *p* < 0.0001. vs. HFD group, n.s: no significance, **** *p* < 0.0001.

Given that a significant proportion of AS patients experience impaired liver and kidney function after taking anti-AS drugs, and some were even found to have changes in organ morphology, we first monitored liver and kidney function and organ tissue morphology in ApoE^−/−^ mice. Biochemical analysis of serum markers revealed that HFD increased CREA levels but did not affect ALT, AST, or UREA levels (Figure 4A–D). According to HE staining results, HFD significantly increased the number of lymph nodules in the spleen but had no effect on the organ morphology of the heart, kidney, and lung (Figure 4E–H). Although DB treatment did not normalize the HFD-induced increase in CREA levels, it significantly reduced the number of splenic lymphoid follicles.

### 3.5. DB Attenuates ox-LDL Internalization in Foam Cells (FCs)

**Figure 5:**
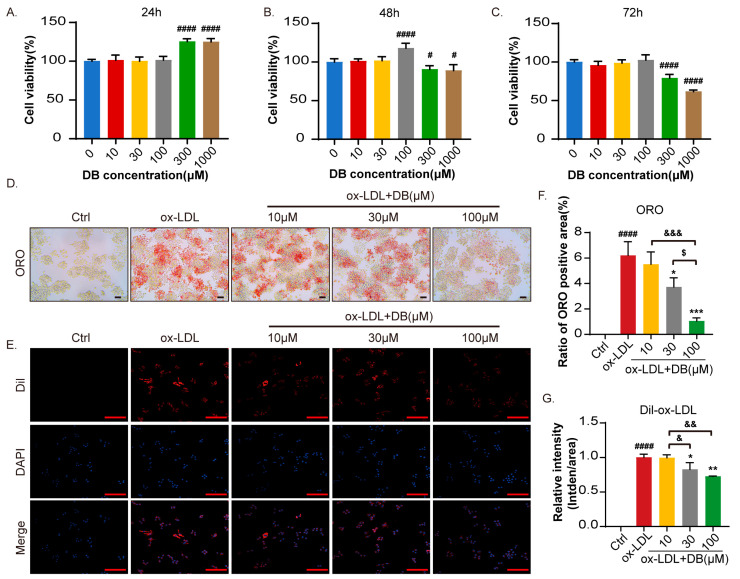
DB treatment inhibited ox-LDL internalization and absorption in FCs. (**A**) CCK-8 assay analysis of cell viability rate of RAW264.7 cells treated with DB of different concentrations for 24 h. (**B**) CCK-8 assay analysis of cell viability rate of RAW264.7 cells treated with DB of different concentrations for 48 h. (**C**) CCK-8 assay analysis of cell viability rate of RAW264.7 cells treated with DB of different concentrations for 72 h. (**D**,**F**) Optical microscopy images of ORO staining and the corresponding quantitative analysis, 200× magnification, scale bar: 100 μm. (**E**,**G**) DiI-ox-LDL level in the FCs and the corresponding quantitative analysis, 400× magnification, scale bar: 100 μm. Data are presented as mean ± SEM vs. 0 μM group or Ctrl group, ^#^ *p* < 0.05, ^####^ *p* < 0.0001 vs. ox-LDL group, * *p* < 0.05, ** *p* < 0.01, *** *p* < 0.001 vs. ox-LDL + 10 μM group, ^&^ *p* < 0.05, ^&&^ *p* < 0.01, ^&&&^ *p* < 0.001 vs. ox-LDL + 30 μM group, ^$^ *p* < 0.05.

To ascertain the appropriate dosage without affecting the vitality of RAW264.7 cells, we primarily employed CCK-8 assay to evaluate the impact of DB on cell viability. As the incubation time prolonged, DB did not exhibit any cytotoxicity at dosages lower than 100 μM. When the concentration exceeded 300 μM, the cell viability presented a biphasic response (Figure 5A–C). Therefore, concentrations of 10, 30, and 100 μM were selected for the subsequent trials. To examine whether DB is involved in FCs formation, ORO staining was used to visualize the lipid droplets in RAW264.7 cells. Compared to the Ctrl group, ox-LDL treatment induced the formation of numerous bright red lipid droplets in RAW264.7 cells, indicating the development of foam cells. DB significantly reduced the size and number of intracellular lipid droplets, and the inhibitory effect could be further amplified by gradually increasing the dosage (Figure 5D,F). Dil-ox-LDL uptake assay revealed that a large quantity of red fluorescence was seen in the cytoplasm and around the nucleus in the ox-LDL group. Consistent with the findings of ORO, DB dramatically reduced the absorption of ox-LDL by RAW264.7 cells (Figure 5E,G).

### 3.6. DB Induces Macrophage Autophagy

**Figure 6:**
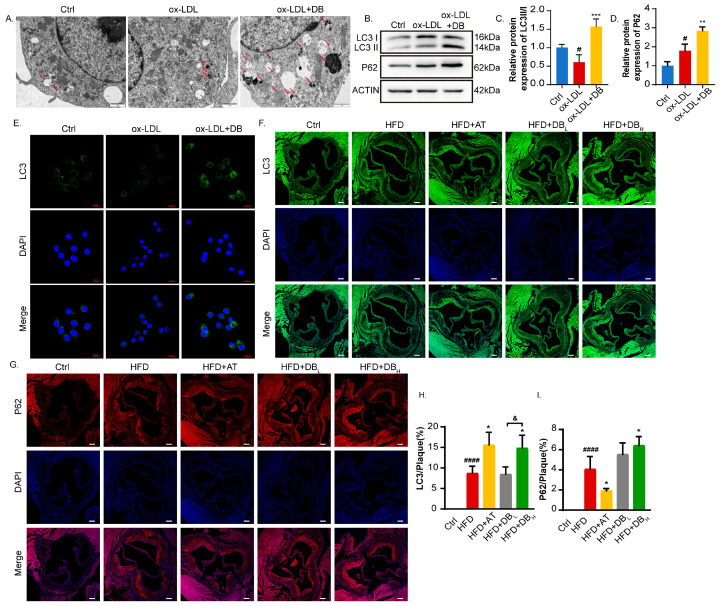
DB treatment restored autophagy both in vitro and in vivo. (**A**) Representative images of TEM revealing the autophagosomes accumulating in the cytoplasm of RAW264.7 cells. The red arrows indicated autophagosomes, 12,000× magnification, scale bar: 1 μm. (**B**) Western blot analysis of LC3 and P62 expression in RAW264.7 cells pretreated with DB for 2 h and coincubated with ox-LDL (100 μg/mL) for 48 h. (**C**,**D**) Quantitative analysis of LC3 II/I ratio and P62 expression in (**B**). (**E**) Confocal fluorescent microscopy images of treated cells stained with LC3 antibody, 600× magnification, scale bar: 10 μm. (**F**,**G**) Confocal fluorescent microscopy images of AS plaques in aortic root stained with LC3 and P62 antibodies, 100× magnification, scale bar: 100 μm. (**H**,**I**) Quantitative analysis of LC3 and P62 expression in (**F**,**G**). Data are presented as mean ± SEM vs. Ctrl group, ^#^ *p* < 0.05, ^####^ *p* < 0.0001 vs. HFD group, * *p* < 0.05, ** *p* < 0.01, *** *p* < 0.001 vs. HFD + DB_L_ group, ^&^ *p* < 0.05. Original images of (**B**) can be found in Supplementary Materials.

Autophagy has been shown to be a therapeutic target for AS. One of the most critical processes in autophagy is the formation of autophagosomes. Thus, the impact of DB on autophagosomes was investigated using TEM. As shown in Figure 6A, ox-LDL treatment did not significantly reduce the number of autophagosomes compared to the Ctrl group. Compared with ox-LDL group, DB greatly increased the number of autophagosomes. When autophagosomes were labeled with microtubule-associated protein 1A/1B-light chain 3 (MAP1LC3/LC3) antibody, ox-LDL significantly decreased the fluorescence of LC3 compared with Ctrl group. In line with the findings of TEM, it was discovered that DB greatly enhanced the production of LC3-positive autophagosomes compared with ox-LDL group (Figure 6E). We further examined two proteins related to autophagy. ox-LDL treatment significantly decreased the LC3-II/LC3-I ratio while increasing sequestosome 1 (SQSTM1/P62) expression. DB treatment significantly increased the ratio of LC3 II/I and P62 expression, which differs from the canonical autophagy process (Figure 6B–D). We continued to examine LC3 and P62 expression in plaques in the aortic root. The results were in accordance with the in vitro findings. DB treatment significantly increased both the LC3 and P62 levels within the plaque areas (Figure 6F–I).

### 3.7. Autophagy Suppression Counteracts Anti-AS Effects and Autophagy Induction Caused by DB

**Figure 7:**
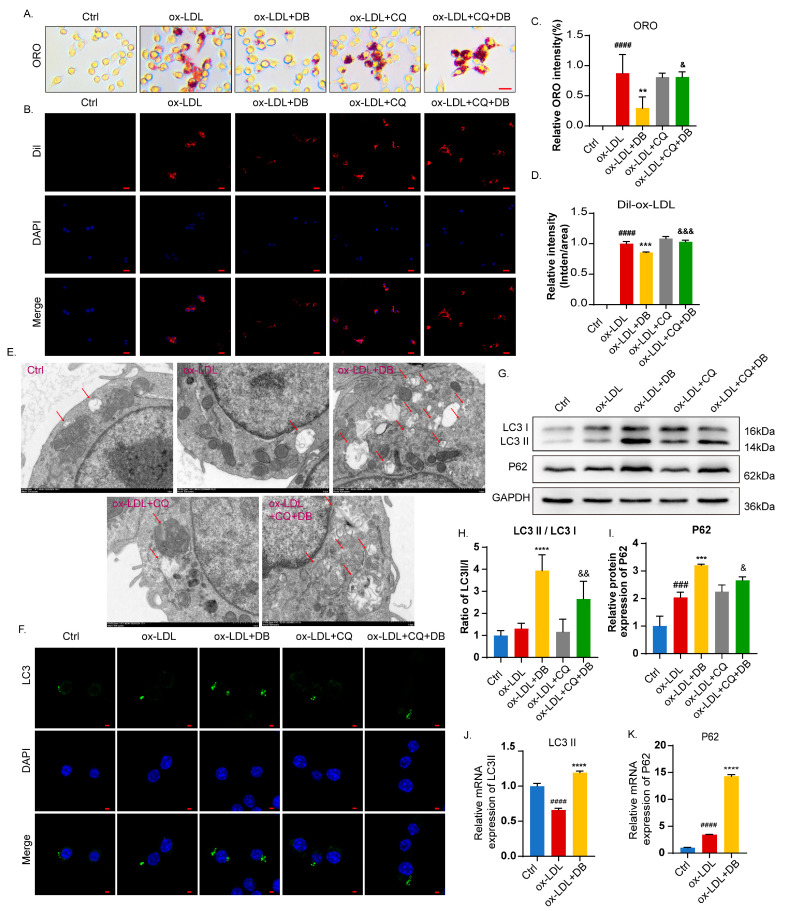
Autophagy inhibition blocked DB-mediated anti-AS effects and autophagy induction effects. (**A**,**C**) Optical microscopy images of ORO staining and the corresponding quantitative analysis, 400× magnification, scale bar: 100 μm. (**B**,**D**) DiI-ox-LDL level in the FCs and the corresponding quantitative analysis, 400× magnification, scale bar: 20 μm. (**E**) Representative images of TEM reveal the autophagosomes accumulating in the cytoplasm of RAW264.7 cells. The red arrows indicated autophagosomes, 10,000× magnification, scale bar: 1 μm. (**F**) Confocal fluorescent microscopy images of treated cells stained with LC3 antibody, 600× magnification, scale bar: 2 μm. (**G**) Western blot analysis of LC3 and P62 expression in RAW264.7 cells pretreated with DB for 2 h and coincubated with ox-LDL (100 μg/mL) for 48 h. (**H**,**I**) Quantitative analysis of LC3 II/I ratio and P62 expression in (**G**). (**J**) Relative mRNA levels of LC3 II in RAW264.7 cells pretreated with DB for 2 h and coincubated with ox-LDL (100 μg/mL) for 48 h. (**K**) Relative mRNA levels of P62 in RAW264.7 cells pretreated with DB for 2 h and coincubated with ox-LDL (100 μg/mL) for 48 h. Data are presented as mean ± SEM vs. Ctrl group, ^###^ *p* < 0.001, ^####^ *p* < 0.0001 vs. ox-LDL group, ** *p* < 0.01, *** *p* < 0.001, **** *p* < 0.0001 vs. ox-LDL + CQ group, ^&^ *p* < 0.05, ^&&^ *p* < 0.01, ^&&&^ *p* < 0.001. Original images of (**G**) can be found in Supplementary Materials.

To further elucidate whether the in vitro anti-AS effect of DB is through autophagy, the autophagy inhibitor CQ was employed in the experiment. Compared to the ox-LDL group, the addition of DB significantly inhibited the foam cell formation and the uptake of the ox-LDL by RAW264.7 cells, while the addition of CQ did not produce such effects (Figure 7A–D). Compared with the ox-LDL + DB group, the addition of CQ abolished the aforementioned effects, indicating that DB exerts its effect via autophagy induction. We then observed the effect of CQ on autophagy index. Compared with the ox-LDL group, DB significantly increased the number of autophagosomes, the ratio of LC3 II/I, and the P62 expression, while CQ did not alter the effects caused by ox-LDL, indicating ox-LDL impaired autophagy in cells. Compared with the ox-LDL + DB group, the addition of CQ did not promote autophagosome accumulation or increase the ratio of LC3 II/I or P62 expression, while CQ reversed the effects brought by DB (Figure 7E–I). To investigate whether DB regulates autophagy by promoting mRNA translation into protein, we employed qPCR to assess the effect of DB on LC3B and P62 mRNA expression. Results showed that DB significantly increased the LC3B and P62 mRNA expression (Figure 7J,K).

## 4. Discussion

**Figure 8:**
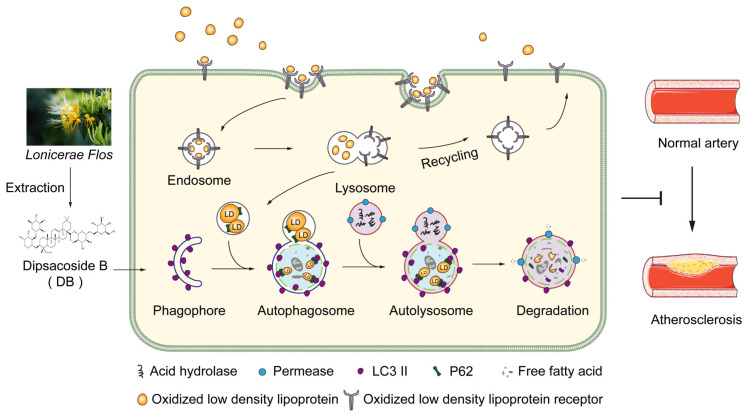
Mechanism of action of DB against atherosclerosis. Ox-LDL uptake by ox-LDL receptors contributes to the autophagy dysfunction. By promoting autophagy process, DB facilitates the encapsulation of lipid droplets into autophagosomes, which then fuse with autolysosomes. Here, acidic hydrolases decompose the lipid droplets into free fatty acids. The free fatty acids are recycled by the body, thus inhibiting the formation of plaques within the intima.

In this work, we demonstrated that DB reduced AS lesions and improved lipid metabolism without compromising hepatic and renal function in AopE^−/−^ mice. In the meantime, it attenuated ox-LDL internalization and promoted autophagy in FCs, as shown in Figure 8. Taken together, our results shed light on the potential mechanism behind DB-induced autophagy and raise the possibility of DB as a promising early-stage treatment for AS.

Recently, many different kinds of natural products have been discovered to exert effects on the treatment of AS [38]. As a class of predominant plant secondary metabolites, oleanane-type pentacyclic triterpenoid saponins are widely distributed in nature and have a variety of pharmacological activities [39]. This kind of saponin has been reported to show excellent properties in the treatment of AS. It was reported that Platycodin D inhibited ox-LDL-induced increase in MDA excretion and monocyte adhesion to endothelial cells, and promoted NO release in human umbilical vein endothelial cells [40]. Afrocyclamin A was found to inhibit the proliferation of VSMCs, reduce the production of pro-inflammatory cytokines, and improve the activity of antioxidative enzymes through the p38-MAPK signaling pathway [41]. In an ApoE^−/−^ mice model, Araloside C protected against AS by reversing the M1 polarization to the M2 phenotype, which was achieved by augmenting Sirt1-mediated autophagy [35]. From the structure-activity point of view, DB shares a parent nucleus with the aforementioned compounds, suggesting that DB could also have comparable anti-AS pharmacological effects. Thus, we first evaluated whether DB affects the progression of AS. In our study, DB treatment did reduce the plaque area, as proved by smaller lesion sizes at different sites of the aorta. The rupture of unstable plaque is a significant starting factor in the onset of ACS [42]. The key pathological hallmarks of unstable plaque are macrophage infiltration, a thin fiber cap, a large lipid core, etc. [43]. Our data showed that the necrotic core, MMP-9, and CD68 expression in the plaque was reduced while collagen content was increased after DB treatment, suggesting that DB promotes more stable plaques. In addition, DB treatment significantly affected mice’s blood lipids, as proved by lower levels of TG, TC, and LDL in the plasma, and it also inhibited lipid deposition in the liver. Taken together, these findings indicate that DB treatment strongly regulates lipid metabolism. Then, we observed the influence of DB on liver and kidney function, main tissues, and organ morphology. The results showed that HFD significantly caused the increase in UREA, but had no significant effect on ALT, AST, or CREA. Compared with the HFD group, DB treatment had no discernible impact on UREA level, indicating that DB serves its activities without interfering with the regular functions of the liver and kidneys. In AS lesions, the spleen may act as a reservoir of monocytes infiltrating AS plaques, while lymph node formation is a manifestation of the body’s anti-inflammatory response [44]. HE staining showed that HFD significantly increased the formation of lymph nodules in the spleen, but no impacts were observed in other organs. The size and number of lymph nodules were significantly decreased after DB treatment, indicating that DB can improve the chronic inflammatory response caused by HFD.

In the development of AS lesions, macrophages are the primary culprits. They phagocytose ox-LDL in the subintima, generating FCs and deposits, and subsequently secrete proinflammatory mediators that cause blood vessel inflammation to persist over time and encourage the retention of lipoproteins at plaque sites [45]. Based on previous research about the pharmacological functions of DB [25,26,27], we further investigated the effects of DB on the cell viability of RAW264.7 cells. The results showed that DB significantly promoted cell viability within 24 h when the concentration exceeded 100 μM. With the incubation time prolonged, DB significantly inhibited the cell viability when the concentration exceeded 100 μM. Based on these results, concentrations of 10, 30, and 100 μM were selected for subsequent experiments. ox-LDL, a potent oxidizer, was coincubated with RAW264.7 cells to construct a FC model in the in vitro experiment. It was observed that DB significantly inhibited the accumulation of ox-LDL and reduced the uptake of lipids by macrophages, indicating that DB can significantly inhibit the formation of FCs. Additionally, we found that 100 μM was the optimal concentration and was chosen for the next investigation into the mechanism.

Autophagy, as its name implies, is a membrane-mediated biological process involving the phagocytosis and transport of cytoplasmic components to lysosomes for degradation. It consists of four main processes: the initiation of autophagy, the formation of autophagic vesicles, the formation of autophagosomes, and the fusion and degradation of autophagic lysosomal content [46,47]. It is reported that promoting macrophage autophagy can delay the occurrence and progression of AS by reducing lipid absorption, cell apoptosis, and vessel inflammation, and increasing cholesterol efflux and exocytosis [48,49]. Hence, active strategies aimed at promoting macrophage autophagy have far-reaching implications for the treatment of AS. One of the best indicators of autophagy is the considerable production of autophagosomes. MAP1LC3, also known as LC3, is a specific marker of autophagosome formation that is incorporated into the inner and outer membranes of autophagosomes during the process of autophagosome assembly [50]. The primary role of the multifunctional ubiquitin-binding protein SQSTM1, commonly referred to as P62, is to bind ubiquitin proteins and transfer them to autophagosomes or proteasomes for degradation [51]. Both of the two proteins are frequently employed to signify autophagy’s steady progression. It was discovered that DB significantly promoted the formation of autophagosomes, as evidenced by more autophagosomes or LC3-labled autophagosomes observed under TEM or CLSM, respectively. Observation of the changes in autophagy-related proteins revealed that DB significantly increased the proportion of LC3II/I and promoted the expression of P62, which was in contrast to the conventional belief that P62 was negatively correlated with autophagy activity [52]. Further examination of changes in expressions of LC3 and P62 on the plaques in the aortic root was consistent with the in vitro research. Nonetheless, investigations in recent years have suggested a favorable correlation between P62 and autophagy activity [53,54]. Combined with the findings that DB had a significant inhibitory effect on foam cell formation in vitro, we speculated that the rise in P62 expression could be positively related to the elevated autophagic activity. Therefore, we employed autophagy inhibitor CQ to observe whether the effects of DB on foam cells and autophagy were reversed after autophagy flux was blocked. Additional analysis of autophagosome formation and autophagy-related protein expression revealed that, compared with the ox-LDL+DB group, autophagosomes significantly decreased, and the LC3II/I ratio and P62 expression significantly reduced after the addition of CQ. The findings demonstrated that CQ countered DB’s inhibitory impact on intracellular lipid uptake and accumulation, indicating that DB exerts its role by promoting autophagy. Although this phenomenon contradicts the traditional view [55], the aforementioned effects of DB were abrogated by the addition of CQ, indicating a regulatory loop. Since autophagy is a dynamic equilibrium process, it is necessary to monitor changes in autophagy flux over time [56]. According to research [57], P62 can be regulated at the transcriptional level to encourage more protein translation. The amount of translated P62 protein outpaced the amount of those degraded by the autophagy process, resulting in the upregulation effects. Thus, we used the qPCR technique to explore the mRNA expression of LC3 and P62. The results showed that DB significantly increased the expression of LC3 II and P62 mRNA, suggesting that the effect of DB on autophagy proteins may be regulated at the transcription level.

Additionally, there are still a number of issues that need to be further elucidated. Firstly, we have demonstrated DB suppressed the accumulation in RAW264.7 cells. Whether these suppression effects were achieved by influencing scavenger receptors such as LOX-1, CD36, needs to be tested. Then, it is well known that there is more VLDL than LDL in the plasma of ApoE^−/−^ mice, which has a different profile from that of humans [58]. We have observed that DB significantly reduces LDL levels, and further experiments are needed to evaluate its effect on VLDL profiles. Literature indicates that pentacyclic triterpene oleanane-type saponins regulate blood lipid levels by inhibiting the intestinal absorption of dietary cholesterol [59], reducing inflammation to enhance endothelial function [60], and mitigating oxidative stress damage [60]. Based on our experimental design, we hypothesize that DB improves lipid profiles primarily by inhibiting the absorption of dietary cholesterol in the intestines. To explore the underlying mechanisms, we will employ high-throughput sequencing technology to identify the potential pathways involved.

## 5. Conclusions

In conclusion, DB demonstrated significant potential in the treatment of AS by effectively reducing plaque formation and enhancing lipid metabolism. DB inhibited the uptake of oxidized ox-LDL in foam cells and promoted autophagy, leading to improved lipid degradation. Importantly, DB’s administration did not adversely affect liver or kidney function, highlighting its safety for therapeutic use. The increase in autophagy markers, such as LC3 and P62, further supported DB’s role in enhancing autophagic activity, making it a promising candidate for early-stage atherosclerosis treatment.

## Figures and Tables

**Table 1:** Primer sequences used for qRT-PCR.

Primer Name	Sequence
Mouse-*MAP1LC3B*-F	GAGACATTCGGGACAGCAAT
Mouse-*MAP1LC3B*-R	CTATGTGGGTGCCTACGTTC
Mouse-*P62*-F	CCACCCCCTTTGTCTTGTAGT
Mouse-*P62*-R	GCCTGAAAAGGCATCACACAT
Mouse-*β-Actin*-F	GTGACGTTGACATCCGTAAAGA
Mouse-*β-Actin*-R	GCCGGACTCATCGTACTCC

## Data Availability

The data presented in this study are available on request from the corresponding author.

## Supplementary Materials

The following supporting information can be downloaded at: https://www.mdpi.com/article/10.3390/biom14101226/s1, Original images of Figure 6B and Figure 7G can be found in supplementary materials.
