# Supplementary figures and images for: Dipsacoside B Attenuates Atherosclerosis by Promoting Autophagy to Inhibit Macrophage Lipid Accumulation
Source: Biomolecules. 2024 Sep 27;14(10):1226. doi: 10.3390/biom14101226 (PMC11506285; doi:10.3390/biom14101226)

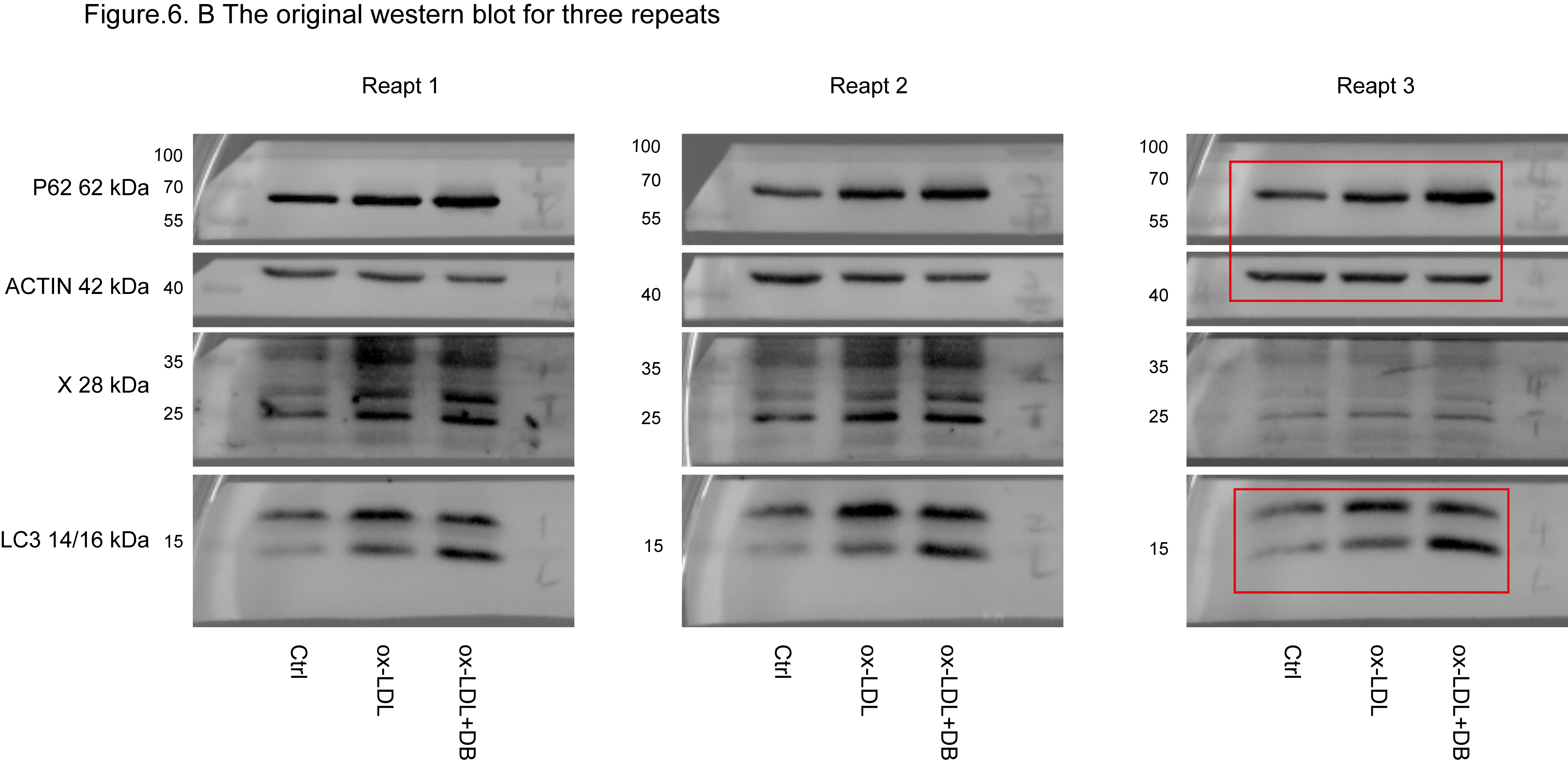

Supplement: Supplementary file 1 [file biomolecules-14-01226-s001.zip › Original_Images_for_Blots/Original Images for Figure 6B.tif]

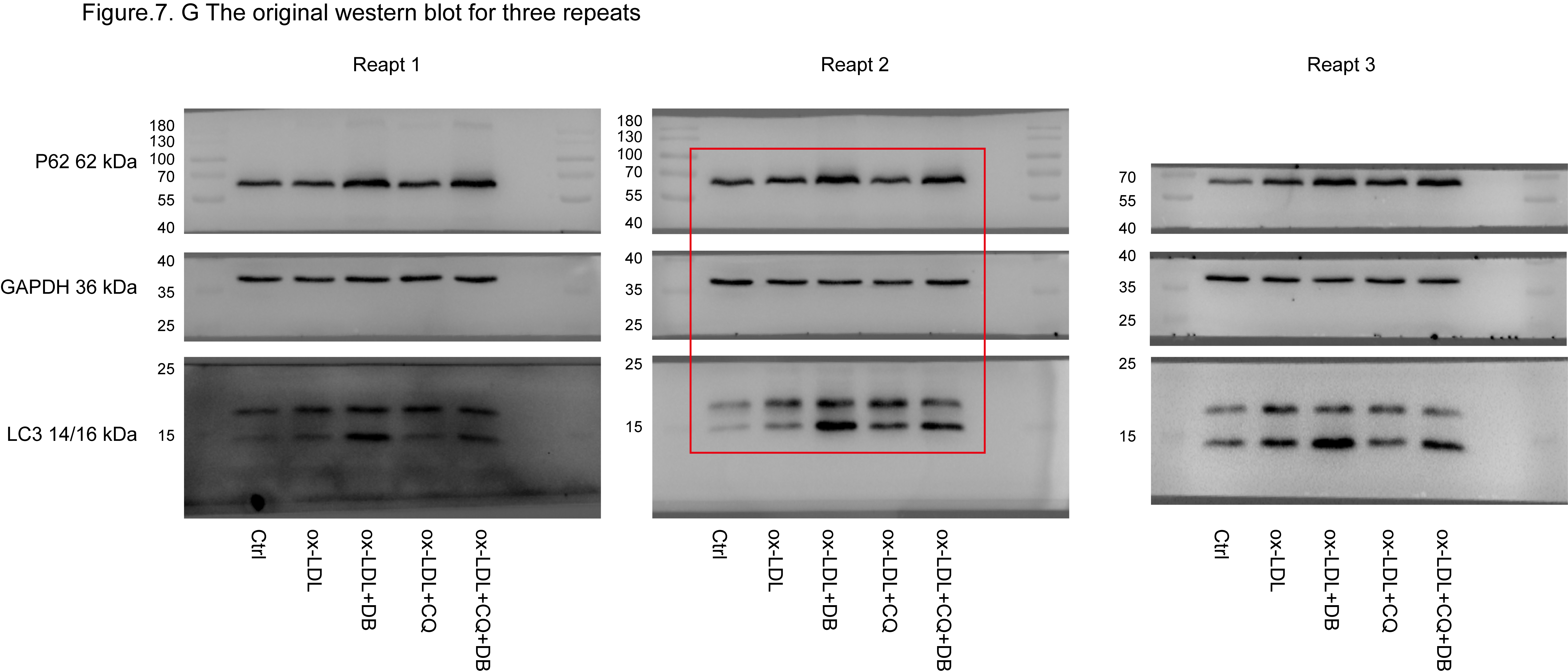

Supplement: Supplementary file 1 [file biomolecules-14-01226-s001.zip › Original_Images_for_Blots/Original Images for Figure 7G.tif]
